# Designing Front-of-Package Labels to Inform Consumers and Encourage Healthier Food Choices in Bangladesh: A Qualitative Study

**DOI:** 10.3390/nu16233989

**Published:** 2024-11-21

**Authors:** Lindsey Smith Taillie, Ahmad Khairul Abrar, Ummay Afroza, Jubaida Akhtar, Violet Noe, Nicole Ide, Nora Abdel-Gawad, Sohel Reza Choudhury

**Affiliations:** 1 Department of Nutrition, Gillings School of Global Public Health, Chapel Hill, NC 27599, USA; taillie@unc.edu; 2 Carolina Population Center, University of North Carolina at Chapel Hill, Chapel Hill, NC 27516, USA; violetn@email.unc.edu; 3 Department of Epidemiology and Research, National Heart Foundation Hospital and Research Institute, Plot-7/2, Section-2, Mirpur, Dhaka 1216, Bangladesh; abrar.bd@gmail.com (A.K.A.); afrozatamanna.bd@gmail.com (U.A.); jubaidasa@yahoo.com (J.A.); 4 Resolve to Save Lives Inc., 1520 Belle Vista Blvd Suite 4036, Alexandria, VA 22307, USA; nide@rtsl.org (N.I.); nabdelgawad@rtsl.org (N.A.-G.)

**Keywords:** front-of-package labeling, warning label, GDA, packaged foods, ultra-processed foods, non-communicable diseases, food policy, Bangladesh

## Abstract

**Background/Objectives:** Front-of-package labeling (FOPL) policies are a useful strategy to inform consumers about foods high in nutrients of concern, but little is known about what type of label works best in Bangladesh, a country with increasing levels of unhealthy food intake and diet-related diseases. **Methods:** We conducted 10 focus groups with men and women in rural and urban Bangladesh (*n* = 76). Using a semi-structured discussion guide, we asked consumers for their perceptions of the healthfulness of nutrients and foods, two common FOPLs (a color-coded guideline daily allowance [GDA] label and a warning label), and different visual elements of the warning label (e.g., shape, icon, text). **Results:** Participants understood the health harms of sugar and salt consumption but were less clear on saturated fat. Both FOPLs were perceived as helpful for identifying unhealthy foods, but the warning labels were perceived as easier to understand and more likely to influence behaviors than the GDA. Regarding the design of warning labels, participants perceived warning devices, holding straps, and octagonal shapes as effective but had mixed reactions to which icons or textual statements were most effective. **Conclusions:** FOPLs are likely to facilitate Bangladeshi consumers’ ability to identify unhealthy products. Further research is needed to understand the impact on food choices as well as the most effective design in this population.

## 1. Introduction

Across the globe, there is mounting concern regarding the consumption of highly processed foods and its link with obesity, hypertension, and other non-communicable diseases (NCDs). In Bangladesh, despite the continued high prevalence of undernutrition [1,2,3,4], the prevalence of obesity is rising [3,5,6], with a nearly three-fold increase among women and a 1.5-fold increase among men between 2004 and 2018 [3]. NCDs now represent 14 of the 20 leading causes of death in Bangladesh [3,7,8]. Along with this increase in obesity and NCDs, packaged processed food consumption has become highly prevalent. Prior studies indicate that 61–83% of Bangladeshi consumers report consuming at least one packaged food over a 24 h period and, on average, consume packaged food 8.6–14.6 times per week [9,10,11]. Not only is consumption of packaged processed food highly prevalent, but other unhealthy food behaviors, such as snack food consumption and visiting fast food restaurants, are frequent [12,13]. Data also demonstrates that Bangladeshi consumers’ diets are high in nutrients linked to unhealthy dietary patterns. Dietary salt intake among Bangladeshi adults is almost double the World Health Organization’s (WHO) maximum recommended level of 5 g per day, with intake estimates ranging from 6.7 to 9 g/day [14]. Furthermore, data from the 2011 and 2018 Bangladesh Integrated Household Survey found that over 70% of participants had excessive carbohydrate intake and that fat intake increased by 57–68% in children and 22–40% in adults [15].

The nutrition transition in Bangladesh calls for population-level strategies to prevent continued increases in obesity and NCDs. Mandatory nutrition labeling is a foundational policy to improve diets since it affects the entire food supply, ensures consumers have access to the information they need to make decisions, and can help them make healthier choices [16,17,18,19]. In addition to being difficult to understand, barriers to using nutrition labels include illiteracy, lack of awareness, and distrust [20,21,22,23]. Consumers also prioritize other information about the food, such as brand names and expiration date, over nutrition information [24,25]. For example, a survey in south India found that while 85% of consumers looked at the brand name and 80% looked at the expiration date, less than 40% checked nutrition information on food labels [25]. This raises the question of Bangladesh consumers’ current understanding of nutrients of concern, which is essential for informing population-level action to raise nutrition knowledge and NCDs awareness.

A second critical question relates to how to most effectively communicate nutrition information to consumers. Front-of-package labels (FOPLs) are recommended by the World Health Organization and others as a key policy strategy to quickly and easily help consumers make healthier food choices in a complex food environment [26,27,28,29,30,31]. However, there are many different types of FOPLs, ranging from the numerical guideline daily allowance to summary measures like Nutri-Score to color-coded options like traffic lights to simple black and white warning labels. It is not currently clear which FOPL will be most effective in the Bangladeshi context with regard to visibility, credibility, comprehension, and ability to help consumers quickly and easily identify when a product is high in nutrients of concern. In addition, for warning labels in particular, key questions related to what design elements (e.g., shape, icons, text) will be best understood by consumers. An understanding of consumers’ responses to FOPL types as well as design elements is critical for advocates to be able to advance an evidence-based FOPL policy in Bangladesh.

The objectives of this research were to use focus group discussions with diverse Bangladeshi residents to understand their perceptions of nutrients of concern and how they are linked to NCD risk, assess consumers’ reactions to different FOPL types, and assess the interpretation of design elements, particularly for warning labels.

## 2. Materials and Methods

This study was approved by the Institutional Review Board, Ethics Review Committee, of the National Heart Foundation Hospital and Research Institute (approval reference number: NHFH&RI/4-14/7/Ad/1969).

Broadly, this study was based on international guidance on best practices for designing FOPL policies [32] as well as previous FOPL design research conducted in other countries [33,34].

### 2.1. Setting

We conducted 10 focus groups between December 2023 and April 2024. The total sample comprised 76 adults aged 18–50 years. Focus groups were conducted in rural/urban areas based on definitions set by the Bangladesh Bureau of Statistics, with 50% of focus groups taking place in urban areas, including Dhaka, Sylhet, and Jamalpur, and 50% of focus groups taking place in rural locations, including rural areas near Dhaka, Jamalpur, Kishoreganj, and Rangpur. Each focus group included 6 to 9 participants and lasted between 59 and 95 min.

### 2.2. Sample

Eligibility criteria included (1) being a Bangladeshi resident; (2) age between 18 and 50 years; and (3) purchasing any packaged processed foods in the last month. The study used a convenience sample [35,36]. The research team recruited research assistants from the preselected study area and trained them on the study design and components, especially the eligibility criteria. The research assistants went house to house and screened potential participants. Eligible participants were briefed about the study and invited to participate. Participants who agreed to participate voluntarily and provided written consent were included in the focus group discussions (FGDs).

Focus groups were stratified by area of residence (urban/rural) and by gender (60% of groups comprised of men). The rationale is that external stakeholders indicated the importance of including representation from these groups for the purposes of informing FOPL policies in Bangladesh and, for gender specifically, men and women have different purchasing behaviors in Bangladesh with regard to packaged foods, with men being more likely to be the primary purchasers within the household.

### 2.3. Label Design

**Figure 1:**
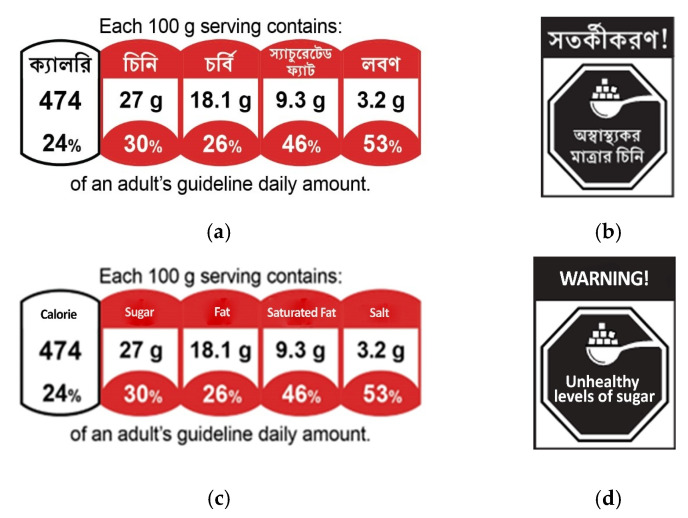
Example front-of-package labels. (**a**) Example GDA label. (**b**) Example warning label for sugar. (**c**) Example GDA label in English. (**d**) Example warning label in English.

FOPLs were selected for testing in the focus groups based on (a) a review of the literature; (b) discussion with global food labeling experts; and (c) interviews with key stakeholders in Bangladesh, including policymakers, scientists, and leaders of non-governmental health organizations. The research team sought to include front-of-package labels that were based on scientific evidence, relevant, and had a greater likelihood of being considered for a future FOPL policy in Bangladesh. Based on these criteria, the labels selected to be included in the study were a nutrient warning label and a color-coded guideline daily amounts (GDA) label. Images of the FOPLs are depicted in Figure 1.

Warning label: the nutrient warning label’s design was based on warning labels currently in use in Chile, Mexico, Colombia, and other countries in Latin America. The label comprised black octagons inside a rectangular holding strap. Each octagon included, for the relevant nutrient, an icon and text (“unhealthy level of sugar”, “unhealthy level of salt”, “unhealthy level of fat”). The rectangle also included a warning device with the text “warning”. A product received a warning label for a given nutrient if it contained sugar, salt, or saturated fat and exceeded nutrient thresholds according to the UK traffic light system (sugar > 22.5 g, salt > 1.5 g, and saturated fat > 5.0 g, all per 100 g of food). The UK Traffic Light System was used as the underlying nutrient profile model because it establishes thresholds for “high” levels of a nutrient as well as medium and low (as used in the color-coded GDA label described below), allowing for a consistent application of the nutrient threshold across all label types [37]. Salt was indicated, rather than sodium, as stakeholder meetings indicated salt was better understood by Bangladeshi consumers, national dietary guidelines refer to salt, and Bangladeshi package labeling laws require salt, not sodium, to be labeled on packages. For fat, even though saturated fat content was used to identify which products should receive a warning label, the term “fat” was displayed on warning labels. This decision was made due to stakeholder feedback that the term saturated fat would be poorly understood, whereas the term fat is more readily understood. Stakeholder and expert feedback revealed that in Bengali, people typically understand “oil” to refer to unsaturated fat and “fat” to refer to saturated fat. Thus, for warning labels, the word “fat” was used for clarity and intended to represent “saturated fat”.

Color-coded GDA label: the color-coded GDA label was based on voluntary GDA labels currently in use by some food manufacturers in Bangladesh and globally. The GDA label included calories, sugar, fat, saturated fat, and salt, including the absolute amounts per 100 g and % of an adult’s guideline daily amount. Each nutrient was color-coded green, yellow, or red for low/medium/high in a nutrient of concern, based on the UK traffic light system [38]. For the color-coded GDA label, both fat and saturated fat were included since pre-existing GDA labels typically include both nutrients.

**Figure A1:**
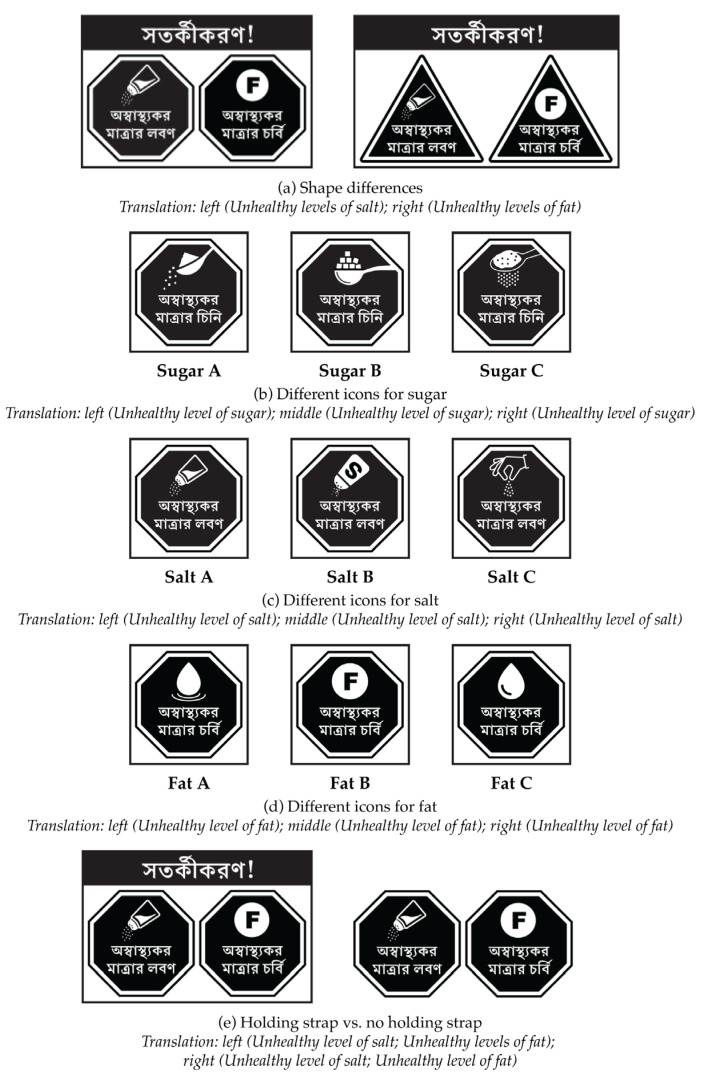
Warning label design elements. (**a**) Shape differences. (**b**) Different icons for sugar. (**c**) Different icons for salt. (**d**) Different icons for fat. (**e**) Holding strap vs. no holding strap.

Because the warning label was the label indicated to be most relevant for local advocacy campaigns on FOPLs, we also tested a set of alternative designs for warning labels, which included different shapes, icons, and holding straps as well as different text (e.g., unhealthy, dangerous, and excess levels of [nutrients of concern]. Images of these labels can be found in Figure A1.

**Figure 2:**
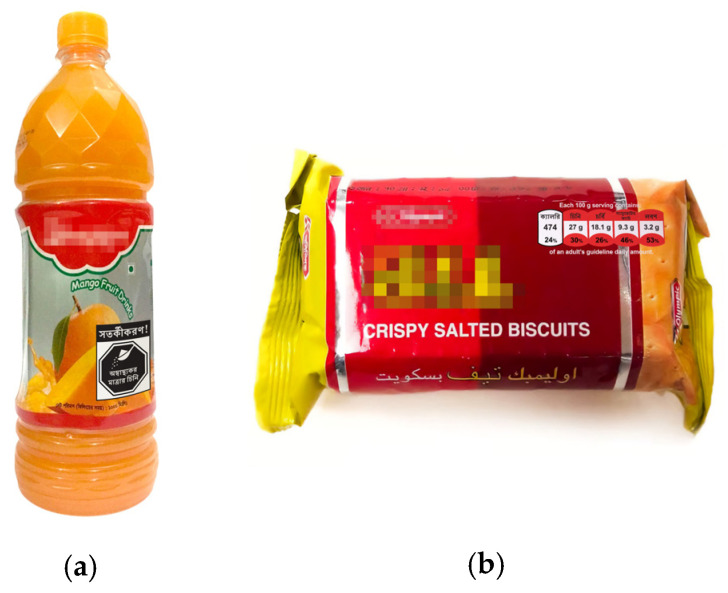
Examples of product mockups with labels. (**a**) Fruit drink with warning label. (**b**) Biscuits with color-coded GDA label.

To show the FOPLs in the context of real food purchasing decisions, we displayed the labels on digital images of real Bangladeshi food products, with product brand information blurred as seen in Figure 2.

Products with four different package shapes were selected based on formative data on popular packaged foods consumed in Bangladesh [39], including instant noodles, potato crackers (chips), biscuits, a fruit drink, and yogurt.

### 2.4. Procedure

After completing an eligibility screener (including demographic information) and informed consent, participants participated in an in-person focus group discussion, which comprised general nutrition questions as well as reactions to FOPLs. Trained moderators used a semi-structured focus group discussion guide prepared by the research team. Focus groups were recorded with participant permission, transcribed, and translated into English.

The guide was developed based on a conceptual framework for how front-of-package labels work, specifically by influencing attention, attitudes, knowledge, and behavioral intentions [40,41], as well as guides previously used to assess consumer reactions to FOPLs in other countries [33,34].

The guide comprised the following sections: (1) perceptions of the healthfulness of different foods and nutrients; (2) perceptions of front-of-package labels; and (3) design elements of warning labels. During the relevant sections, the moderator displayed the product mockups with the FOPLs for participants to observe during the discussion. The full discussion guide is included in Appendix B.

In the first section about healthfulness perceptions, participants were asked about the characteristics of healthy and unhealthy foods. Then, they were asked about their perceptions of foods that were high in sugar, salt, and saturated fat, examples of foods high in these nutrients, and the health effects of consuming foods or drinks high in these nutrients.

In the second section comparing types of FOPL, participants were asked to assess each label’s visibility and memorability, comprehensibility, and potential effectiveness (e.g., if the label would change their attitude towards a product or affect their decision to purchase it). This section also asked participants to compare the warning labels and GDA labels.

In the third section about warning label design elements, participants were asked if each design element (shapes, icons, text options, holding straps, warning devices) was inappropriate or offensive, if they helped with their understanding of the label, and which they thought would be most effective at grabbing people’s attention and reducing purchases of unhealthy foods. We did not evaluate color due to prior research indicating that black labels are better able to stand out from the package and capture attention [42].

### 2.5. Positionality

Characteristics of the research team have the potential to influence the study team’s interactions with participants, observations, and interpretations [43]. Our study team comprised researchers from Bangladesh and the United States. All moderators who interacted with participants were from Bangladesh.

### 2.6. Analysis

We tabulated descriptive statistics on the sociodemographic characteristics of the sample.

For the qualitative analysis, we used a thematic approach, utilizing NVivo 14 to organize and code data. Initial codes were defined based on our theoretical understanding of how front-of-package labels affect behavioral change [40,41,44,45], as well as based on previous qualitative FOPL research [33,34]. These deductive codes were then grouped into main themes, some of which matched questions from the semi-structured focus group guide. After coding transcripts, the research team used inductive analysis to identify additional codes and subthemes, subsequently refining the codebook.

## 3. Results

### 3.1. Descriptive Results

Table 1 includes a description of participants’ socio-demographic characteristics. Most of the participants were between 18 and 34 years old, with about a third with less than secondary school education, a third who completed secondary or higher secondary, and a third who completed college or university, and the majority had children under the age of 16 years. Most of the sample consume packaged food and drinks a few times a week or once a day, with only 14.5% never consuming them and 13.2% consuming them more than once a day.

### 3.2. Perceptions of Healthfulness, Unhealthfulness, and Nutrients of Concern

Table 2 depicts the themes and sub-themes identified in the qualitative analysis.

During the discussion, virtually all participants agreed that their perceptions of whether a food was “healthy” related to whether it contained sufficient nutrients of benefit, including protein, carbohydrates, vitamins, minerals, and fiber. For example, one female participant noted,

“If a food contains all the necessary elements for our body, we can consider it a nutritious food. For example, if a biscuit contains flour, sugar, salt, milk, and some liquid glucose in appropriate quantities, then that biscuit will also be healthy for us.”

Few participants discussed whether a food needed to be “low” or “free” from certain ingredients to be healthy. Only one participant mentioned that to be healthy, a food needed to be free from excess amounts of ingredients or nutrients; a few participants mentioned being lower in fat or oil or not fried, and others mentioned that healthy foods should be preservative-free or “natural”.

Many participants were concerned with food safety; in other words, for a food to be healthy, it should be clean, hygienic, “germ-free”, or within the expiration date, and be free from toxins or chemicals. There was a diversity of perspectives on packaged foods, with one participant mentioning not trusting information on labels on packaged foods, while other participants considered packaged food to be healthy relative to unpackaged foods, in part because packaged foods are free from dirt, dust, or insects. For example, one female participant said,

“By healthy food, we mean packaged foods. Flies and mosquitoes sit, and dust and dirt fall on the unpackaged foods, but the packaged food is safe from these.”

In contrast, participants’ perceptions of unhealthy foods were mainly related to lacking essential nutrients (especially protein) or containing high levels of fat, caffeine, sugar, salt, MSG, and spices/spiciness. A few participants mentioned other attributes like containing preservatives, artificial sweeteners, or artificial colors. Many participants perceived oily or fried foods and fast foods/street foods as unhealthy. Even though some participants thought packaged food was healthy, some regarded it as unhealthy. Specific concerns about packaged food relate to not trusting the manufacturing process. Many also perceived concerns relating to being stale, spoiled, or rotten; being unhygienic, passing the expiration date or not containing an expiration date, or containing toxic chemicals, pesticides, or microbes.

With regard to specific nutrients of concern, virtually all participants understood that excess sugar and sodium are associated with health harms (e.g., obesity, diabetes, dental caries, and heart disease for sugar; hypertension, heart, and kidney diseases for sodium). Participants were also able to identify common packaged products that are high in these ingredients (e.g., sweet biscuits, cakes, soda, ice cream for sugar; chips, nuts, salted biscuits, fried peas/pulses, and puffed rice for sodium). In contrast, most participants were not familiar with the term “saturated fat” and did not understand how it was different from fat (in general). Some had a sense that there were “good fats” and “bad fats”, but were unclear about definitions of these. When asked about sources of saturated fats from packaged foods, common responses included chips, cakes, biscuits, butter, and carbonated beverages.

### 3.3. FOPL Evaluation

During the evaluation of FOPLs, participants assessed the labels’ visibility, memorability, and perceived effectiveness. With regard to visibility, most participants felt that both the warning label and the GDA were visible and memorable, though there were some mixed results regarding the GDA (some felt it was more visible due to its colorful appearance, whereas others did not notice it). Participants noticed the colors and numbers on the color-coded GDA label but had difficulty recalling the labels, whereas the warning label’s black color and the word “warning” helped participants recall the label.

With regard to comprehension, warning labels were generally perceived as easier to understand compared to the color-coded GDA. More specifically, participants thought that the color-coded GDA signified what ingredients were present in the food; many had trouble understanding what the numbers and colors on the color-coded GDA meant, with some participants thinking that the numbers and colors meant that the food was healthy or balanced, while others confused the numbers on the color-coded GDA with an expiration date. For example, one rural female participant thought that the color-coded GDA indicated that all of the necessary ingredients were present in the food in the appropriate amounts (i.e., that the food was healthy). Participants were also confused about the absolute numbers and percents listed on the GDA, with some participants thinking that the absolute values were the proportion of that nutrient in the total amount of food; for example, they believed that 45 g of sugar in a 250 g biscuit pack meant that 45% of the biscuit comprised sugar.

In contrast, most participants felt that the warning label was easy to understand; they understood that the warning label meant that the food contained excess amounts of nutrients of concern and that the food was unhealthy. One female participant from Dhaka explained the difference between the two labels:

“Everyone will understand [the warning label]. It can be understood just by looking at a glance. Only one color is used here. For traffic, we have to understand the three colors on the road. But here, it’s all in one color. My problem there (color-coded GDA) would be figuring out what red, yellow, or green means. But here, it’s black. It means, “It cannot be eaten”. It’s harmful to me. The white letters on black are clearly visible.”

With regard to the target audience, participants were concerned that the GDA would be difficult to understand by the general population, and, in particular, children, the elderly, or those with lower education. With regard to the warning label, most participants felt that it would be understandable by all people across sociodemographic characteristics, though some participants had concerns about whether illiterate people would be able to understand it.

These perceptions about comprehensibility translated to differential findings on the perceived effectiveness of the labels on both attitudes and behavioral change. Some but not all participants felt that the GDA might change their attitudes towards unhealthy food products, but to a lesser extent than the warning label, which most participants felt would change their attitudes due to its black color and clear signaling that a product was unhealthy. Similarly, some participants felt that the GDA might change behavior, but only for those who understand it or if an educational campaign was implemented to help raise awareness. In contrast, most participants felt that the warning label would be more likely to get them to reduce or stop buying unhealthy products because of its attention-grabbing nature and signaling that the product is unhealthy. Indeed, when the two FOPLs were compared head-to-head, the majority of participants reported that the warning label vs. the GDA was more helpful for consumers to identify unhealthy foods, stood out more, and was more likely to discourage purchasing of these foods.

Cross-cutting concerns about the FOPLs included believability. Some participants were skeptical, stating they do not believe any information on packages or that the information might be inauthentic. One male participant from urban Dhaka explained:

“When we go to buy packaged food, the details are on the label. But I don’t read those. Because I know that they may be correct or not. Because I don’t trust labels.”

### 3.4. Warning Label Design Elements

The last part of the focus groups looked at warning labels with different design elements. In general, participants felt that icons were attention-grabbing and helped with understanding the label and that the icons would be especially helpful for people with illiteracy or children. However, there was a perception that people would need to learn what the icons meant (somewhat for sugar and especially for fat), and thus a mass media campaign would help people recognize and remember the icons.

With regard to specific icons for sugar, most of the participants found icon A (a spoon with a mound of sugar granules spilling over) the easiest to understand because it depicts a common way of using sugar, and because the overspilling represents excess, whereas icon B (a spoon with sugar cubes) was least preferred since sugar cubes are uncommon. Regarding the icon for salt, participants understood all the icons well. Most of the participants preferred icon C (a pair of fingers pinching salt), as salt is still commonly added to food with a finger pinch during cooking and at the table. Moreover, most of the participants mentioned a very popular television commercial on oral rehydration salt preparation where salt had been added with a finger pinch. Many participants also liked icons A and B as well (both salt shakers; the “B” salt shaker contains the letter S) due to the increasing popularity of using a salt shaker, but there were mixed opinions on the presence of the use of the letter ‘S’ to indicate salt. Specifically, there was concern that the presence of an English alphabet letter would not be suitable for all groups of people. There were also mixed opinions about the best icon to denote fat, though the majority of participants chose icon A (a drop of oil making a ripple) as they were relatively familiar with it. Many of the urban and literate participants preferred icon B (a circle containing the letter F for fat), but others opposed it with a concern that denoting fat by the English alphabet letter ‘F’ would not be well understood by the general population.

With regard to shape, participants felt that both the triangle and the octagon could symbolize “warning”. However, there was some confusion around the octagon because several participants interpreted it as a circle. At the same time, participants were confused by the triangle-shaped warning because it is very similar to the triangle-shaped logo used by the Bangladesh Standards and Testing Institution (BSTI) to indicate product approval. Participants felt that the octagon would be more eye-catching because it is less familiar (i.e., more novel) than the triangle shape, due to the triangle shape already being in frequent use.

All participants preferred labels with the holding strap because the white background helped better visualize the warning label design against the colorful background of the food packages.

Participants had mixed responses to text options “dangerous”, “excess”, and “unhealthy” [levels of a nutrient of concern]. While most participants felt that “dangerous” was the strongest option and most likely to be effective, they worried it would not be accepted by the food industry and may be perceived as too aggressive. In contrast, concerns about “unhealthy” or “excess” were that these might not be understood or would be more easily ignored. The majority of participants felt that the word “warning” and exclamation points worked well and that the holding strap was important to make the warning stand out from the package, be attention-grabbing, and be well understood.

## 4. Discussion

This focus group study of Bangladeshi adults found a pattern of results that suggest that an FOPL could help consumers more easily identify unhealthy packaged foods, but that there are several key considerations for designing an effective FOPL for this population. For example, most consumers felt that “healthy foods” were those that contain sufficient nutrients of benefit, whereas there was relatively low awareness that excess nutrients of concern could make a food unhealthy. Thus, an FOPL that communicates about the excess content of sugar, salt, and fat could help consumers more readily identify unhealthy foods. In general, between a color-coded GDA label and a warning label, participants felt that the warning label would be more understandable across population groups and more likely to influence behavior. However, designing an effective warning label in Bangladesh will also require overcoming key barriers, like consumers’ overall low trust in food labels, as well as identifying which warning label designs can effectively communicate across diverse populations, particularly people with illiteracy.

In this study, Bangladeshi consumers’ perceptions of healthy foods were related to whether a particular food contains necessary nutrients (e.g., protein, carbohydrates, vitamins) or ingredients (e.g., flour, sugar, milk), as well as whether a food was “safe”, or, in other words, clean, free from germs, not expired, and free from chemicals. This prioritization of food safety is similar to results in other low-and-middle-income countries, including in South Asia [46]. In studies in both Myanmar and Vietnam, consumers expressed fear regarding consuming fruits and vegetables due to concerns about chemicals used during production [47,48,49] and shopped for vegetables less frequently when food safety was reported as the main criterion for vegetable purchases [48]. Among women participating in focus groups in Myanmar, the increased prevalence of diabetes and hypertension was correlated with food safety concerns rather than with changes in the nutritional qualities of food via changes in the food supply [47]. In another study in Ecuador, concerns related to food safety were as prevalent or more prevalent than concerns related to nutrients among mothers of young children [50].

Relatedly, there were mixed perceptions as to whether packaged foods were healthy or unhealthy, with some participants feeling that they were healthy because the packaging protects the food from germs or other contaminants, while others felt that they were unhealthy because they do not trust the manufacturing process or may have passed the expiration date. These results are similar to findings in other qualitative studies in which perceptions regarding packaged foods are mixed. In studies conducted in Indonesia, Iran, and Ethiopia, participants associated packaged foods with increased safety and cleanliness [51,52,53], while other studies in Brazil and Mexico found that participants expressed concerns about loss of freshness in packaged foods and distrust in expiration dates [54,55]. There was also mixed awareness that foods high in sugar, salt, and fat were unhealthy (or make a product less healthy). Taken together, the diverse and sometimes conflicting perceptions of what makes a food healthy or unhealthy suggest that clear front-of-package warning labels indicating when foods are high in nutrients of concern could help consumers cut through the noise and more easily determine when a food is unhealthy.

When participants evaluated the color-coded GDA and the warning label FOPLs, the warning labels were consistently easier to understand than the GDA labels. Consumers’ understanding of the color-coded GDA labels was highly variable, with some participants thinking that they signified that a product was healthy or were communicating about the ingredients, and many were confused about what the numbers and colors meant. These results are consistent with other international research on GDA labels or traffic light labels (which use a similar color scheme as the color-coded GDA labels used in this study), which show that when compared to GDA labels, warning labels are more effective at enabling correct identification of products as unhealthy [28,56,57,58,59], discouraging purchase and consumption of unhealthy products [56,60,61], lowering perceived cognitive workload [58], and increasing consumer confidence in ability to choose healthy products [62]. In contrast, participants consistently understood that the warning label signaled that a food had excess amounts of nutrients of concern. Similarly, participants felt that the GDA would change behavior only for those who understood it, whereas the warning label was more likely to help most of the population reduce their purchases of unhealthy products. Comparing the labels, participants felt that the warning label stood out more, was more helpful for identifying unhealthy foods, and was more likely to discourage purchases, all important steps on the pathway from label exposure to behavioral change [40].

Although warning labels appeared more promising than color-coded GDA labels, several key barriers remain. First, participants expressed overall low levels of trust in the information conveyed on food labels. Other studies have found that a reference to a credible authority (e.g., the Ministry of Health or another regulatory agency) can help improve trust in and credibility of FOPLs [63,64,65,66]; however, it is unclear whether this would also be the case in Bangladesh and requires future research. Moreover, participants expressed some concerns over whether the warning labels, which include both text and icons, would be interpretable by low-literacy populations. These results suggest that it would be important for an FOPL regulation to be accompanied by a government-sponsored mass media campaign to help consumers, particularly illiterate consumers, understand what the labels mean. Such a campaign could also potentially address trustworthiness by providing transparency about how the labels are implemented and how the nutritional contents of products are verified.

Regarding design elements for the warning label, participants consistently felt that the warning device and holding strap were effective for making the label grab people’s attention and facilitate understanding. In addition, the octagon was perceived to signal danger and be less confusing than the triangle due to the triangle’s use in other Bangladeshi public communications. However, there was not a consistent icon or textual statement that stood out as most effective. For example, the icons appeared to be helpful in aiding understanding, but the inclusion of letters in some of the icons (like “S” for salt) enhanced understanding for some consumers but not for others. With regard to text, participants felt that the word “dangerous” would be most effective but may not be feasible to implement because it would likely trigger significant industry pushback. On the other hand, participants felt that terms like “unhealthy” or “excess” may not be understood by all. While the results of this study suggest that the warning device, holding strap, and octagon should be used to design a warning label, further design testing is needed to identify the optimal icon and textual statements.

This research has several implications for informing policy. First, the results suggest that front-of-package labels could be an effective strategy for informing the population about which products are unhealthy and that warning labels would be more likely to be effective than color-coded GDA labels. However, given the qualitative nature of these results, future experimental research to test the impact of warning labels vs. color-coded GDAs would be useful to provide additional evidence about the most effective label type. Second, a lack of knowledge around specific nutrients (e.g., saturated fat), as well as low trust in food labels overall, suggest that educational initiatives such as school-based programs or national media campaigns could be used to maximize the effectiveness of an FOPL system that is focused on identifying foods high in saturated fat, sodium, and sugar.

This study had several limitations. One limitation is that products were shown to individuals as images rather than on real-life packages that consumers were considering for consumption. This may have affected consumers’ perceptions (e.g., they are not considering the cost or taste of the product). Future research that includes a more realistic food choice scenario would be useful for better understanding how an FOPL is likely to impact food purchasing behaviors. A second limitation relates to ways in which the warning label design differed from the color-coded GDA; the warning label used only the word “fat”, whereas the color-coded GDA used both “fat” and “saturated fat”. Moreover, the word “fat” had different meanings on the two labels: on the warning label, fat implied saturated fat, whereas on the color-coded GDA, “fat” referred to total fat. The reason for this is because the warning label was designed for simplicity, recognizing that “saturated fat” is not understandable to many people, whereas the color-coded GDA reflected a pre-existing label. However, using the word “fat” instead of “saturated fat” on the warning label may lead to additional confusion given the different health messaging around total vs. saturated fat. Moreover, future testing that seeks to compare the two labels head-to-head in an experimental setting will need to ensure that the nutrient type and language are consistent across label types.

The study also had strengths. One strength of the study was the inclusion of a diverse sample of Bangladesh consumers, including consumers with low educational attainment, women, and participants from both rural and urban areas. This representation is important for ensuring that an FOPL would work well for all Bangladeshi consumers.

## 5. Conclusions

In conclusion, this research on focus groups among Bangladeshi consumers found that consumers have diverse perspectives on what makes foods healthy or unhealthy, but that chief concerns include that a food has sufficient beneficial nutrients, is free from germs or toxins, and does not contain high levels of nutrients of concern. Results suggested that front-of-package labels would help consumers more easily identify unhealthy foods and that warning labels were perceived as more effective than color-coded GDA labels. Among warning labels, design elements like warning devices, holding straps, and octagons were perceived as effective. However, more research is needed to understand other design elements, like icons, and to test the impact of different front-of-package label types on consumer behavior.

## Figures and Tables

**Table 1:** Descriptive characteristics of the sample (*n* = 76).

	N	%
Gender		
Man	46	60.5
Woman	30	39.5
Location		
Urban—Dhaka	16	21.1
Urban—Sylhet	15	19.7
Urban—Jamalpur	7	9.2
Rural—Dhaka	8	10.5
Rural—Jamalpur	9	11.8
Rural—Kishoreganj	14	18.4
Rural—Rangpur	7	9.2
Age group		
18–34	62	81.6
35–50	14	18.4
Highest level of education		
No formal education	3	3.9
Completed primary school	20	26.3
Completed secondary school	15	19.7
Completed higher secondary	13	17.1
Completed college/university	25	32.9
Has children under 16 y		
Yes	53	69.7
Income (taka)		
Urban, mean ± SD (min-max)	31,842 ± 21,378 (9000–100,000)	
Rural, mean ± SD (min-max)	18,763 ± 7401 (10,000–45,000)	
How often do you purchase packaged food and drinks?		
Never or rarely	11	14.5
A few times a week	32	42.1
Once a day	23	30.3
More than once a day	10	13.2

**Table 2:** Themes and sub-themes identified in the qualitative analysis.

Section	Theme	Sub-Themes
Perceptions of healthfulness	Perceptions of healthy foods	Ingredients
		Nutrients
		Level of processing
		Naturalness
		Food safety, cleanliness, and hygiene
		Preparation
	Perceptions of unhealthy foods	Ingredients
		Nutrients
		Level of processing
		Naturalness
		Food safety, cleanliness, and hygiene
		Preparation
		Addictiveness
	Perceptions of sugar, sodium, and saturated fat	Top sources of sugar, sodium, and saturated fat
		Health effects of excess consumption
		Misconceptions
FOPL reactions	Visibility and memorability	Visibility and attention-grabbing
		Memorability
	Comprehension	Ability to understand the purpose of the label
		Meaning of the label
	Other label reactions	Believability
		Cultural appropriateness
		Target population of the label
	Perceived effectiveness	Potential effect on product attitudes
		Potential effect on intentions to purchase
		Perceived benefits and harms
FOPL comparison	FOPL comparison	Selection of which FOPL was perceived as most effective
Alternative design comparison	Label or design preference	Attractiveness and memorability
		Cultural appropriateness
		Informational/understandability
		Perceived effect
		Overall preference

## Data Availability

De-identified data of aggregated codes is available upon request. The data are not publicly available due to confidentiality reasons.

## Appendix B. Full Translated Discussion Guide

**Section 1. Discussion about the healthfulness of foods and drinks**

“First, we are going to talk about what you think about some nutrients, like sugar, and some food and drink products.”

**General Discussion**

1. “First, let’s talk about food in general. When you think of a food that is healthy, what are some of its characteristics? What makes it healthy? When you think of a food that is unhealthy, what are some of its characteristics? What makes it unhealthy?”
2. “Now, we’ll talk about sugar.
  a. When you think about foods that are high in sugar, what are some examples? When you think of drinks that are high in sugar, what are some examples? I’d like you to focus your answers on products that you would buy at a store vs those that you or someone in your household might prepare at home.
  b. Now let’s consider some specific (packaged) foods and drinks that are high in sugar, like (XYZ; show images of a couple of foods and a couple of drinks). Do you think it is good or bad for your health to eat these foods that are high in sugar? Why?
  c. What are some of the health effects of eating too much sugar?”
3. “Now, let’s talk about sodium or salt.
  a. When you think about foods that are high in salt, what are some examples? When you think of drinks that are high in sugar, what are some examples? I’d like you to focus your answers on products that you would buy at a store vs those that you or someone in your household might prepare at home.
  b. Now let’s consider some specific (packaged) foods and drinks that are high in salt, like (XYZ; show images of a couple of foods and a couple of drinks). Do you think it is good or bad for your health to eat these foods that are high in salt? Why?
  c. What are some of the health effects of eating too much salt?”
4. “Now, let’s talk about saturated fat.
  a. What is saturated fat? Where does it come from? (*Answer: saturated fat is a type of fat that has a different structure than unsaturated fat. Saturated fat mainly comes from animal-source foods, whereas most unsaturated fat comes from plants*).
  b. When you think about foods that are high in saturated fat, what are some examples? When you think of drinks that are high in saturated fat, what are some examples? I’d like you to focus your answers on products that you would buy at a store vs those that you or someone in your household might prepare at home.
  c. Now let’s consider some specific (packaged) foods and drinks that are high in saturated fat, like (XYZ; show images of a couple of foods and a couple of drinks). Do you think it is good or bad for your health to eat these foods that are high in saturated fat? Why?
  d. What are some of the health effects of eating too much-saturated fat?”

**Section 2. FOPL Testing**

“Now, I’m going to show you all some images of food packages and their labels, and then ask you some questions about them.”

**Section 2A. Main Label Testing:**
[*Repeat this section for both labels that will be tested]. Then, proceed to show the main image for testing Label #1 (e.g., the 4 products with Label #1). Keep it up for 10 s or so until everyone seems to have seen it clearly. Then turn off the image.*]
Visibility/Memorability

- “Were the labels easily visible? Did it grab your attention? How visible was it? Was it immediately visible or not? Did it catch your eye?”
- “Were the labels memorable? Why or why not?”
- “Can you recall the label for me now? What exactly did it look like? What do you recall of its shape, color? Was there any text in it? What did it say?” [*Without leading their answers, probe respondents’ memory of its shape, color, text, icon,* etc.]
  “Now, I’m going to show you the labels again and ask you more questions about them.” [*You can highlight the specific labels you are talking about*.]

Comprehensibility

- “What did you understand from the labels?
- Is there anything you did not understand about the label or that confused you about it?
- What did the labels tell you about the food and drinks they were on? (probe for if they thought the product was healthy or unhealthy)
- Who do you think these labels are for?
- Did you believe what the label said?
- Is there anything about this label that is culturally inappropriate? Is there anything about it that is likely to be difficult to understand/interpret for Bangladeshi food shoppers?”

Potential Effectiveness

- “If you were at the store and saw these labels on a food or drink package, would they change your attitude toward the product? How?
- If you saw this label on food or drink packages in a store, would it affect your decision to buy that product or not? How would it affect your decision to buy it?”

**Section 2B. Comparative Rating**
*Show a slide that contains both label types (Warning + GDA).*

“Now please look at both labels you have seen today. Does either label stand out for you? What about that label makes it stand out the most? Why do the other labels not stand out as much to you?

Which label do you think would most help you identify that food was unhealthy? Which label would most discourage you from buying unhealthy foods?”

**Section 3. Warning label elements**

“Now, we are going to talk about some of the design elements of the warning label that you saw. We’d like to consider various elements of the label design, and I’d like your views on whether changing it would improve the effectiveness of the label or not. To start off, I’d like you to consider the ICONS or the pictures we’ve used in the label. I’ll show you the label you saw earlier and some alternatives.”

“The following are the elements of the front-of-pack warning label that will be tested. Please familiarize yourself with them so that you may guide the participants effectively.”

EXAMPLE (REPLACE):

“Let’s start with the icon of FAT. Of the options before you:

- Is there anything inappropriate or offensive about the alternatives proposed?
- Do the alterations improve your understanding of the label? That is, does it give you a more accurate understanding of what the label intends to say? How does it do so?
- Does the presence of an icon (compared to no icon) improve your understanding of the label? How does it do so?
- Of the options before you, which one do you think is more likely to be effective in grabbing people’s attention and deterring the purchase of unhealthy food? How does it do so?
  “Now, I’d like us to look at the SYMBOL/HOLDING SHAPE and COLORS we’ve used for the label. [*Moderator, point to the outside shape to ensure that participants have understood exactly what was meant*.] At the same time, I would like us to look at the symbol/holding shape in different colors. Here’s the original label together with an alternative symbol shape”. [SHOW SLIDE] and here are both symbols in different colors. [SHOW SLIDE] [*Moderator to move to the three appropriate slides, in step with the discussion and questions below.]*
  “Now, I’d like us to look at different food products with different symbols in different colors.” [SHOW SLIDE]. Here is another product with all of the symbols in different shapes and in red and black. [SHOW SLIDE].

- Do the alterations improve your understanding of the label? That is, does it give you a more accurate understanding of what the label intends to say? How does it do so?
- Does the presence of the (alternate shape) improve your understanding of the label? How does it do so?
- Does the presence of the (alternate color) improve your understanding of the label? How does it do so?
- Of the options before you, which one do you think is more likely to be effective in grabbing people’s attention and deterring the purchase of unhealthy food?
  “Now, I’d like us to look at the WARNING DEVICES on the label. [*Moderator, ensure that the participants understand what is being referred to.*] I’ll show you the initial label again, and an alternative to the original”. [SHOW SLIDE]
  “Here’s the initial label with the initial warning device. And now here are three alternatives; one with an additional exclamation mark, one without the warning text, and the last one in red as well.” [*Moderator to move to the appropriate slides, in step with the discussion and questions*.]

- Do the alterations improve your understanding of the label? That is, does it give you a more accurate understanding of what the label intends to say? How does it do so?
- Does the presence of the (alternate warning device) improve your understanding of the label? How does it do so?
- Of the options before you, which one do you think is more likely to be effective in grabbing people’s attention and deterring the purchase of unhealthy food? [*Moderator, ensure that each participant’s response is solicited and noted by the research assistant*.]
  “Now, I’d like us to look at the HOLDING STRAP we’ve used around the label. [*Moderator, ensure that the participants have understood what is referred to.*] I’ll show you the initial label again, and an alternative”. [SHOW SLIDE]

[*Moderator to move to the appropriate slides, in step with the discussion and questions below.*]
Here’s the initial label (white background). And now here is an alternative (black background).

- Do the alterations improve your understanding of the label? That is, does it give you a more accurate understanding of what the label intends to say? How does it do so?
- Does the presence of the (alternate holding strap) improve your understanding of the label? How does it do so?
- Of the options before you, which one do you think is more likely to be effective in grabbing people’s attention and deterring the purchase of unhealthy food? [*Moderator, ensure that each participant’s response is solicited and noted by the research assistant*.]
